# Survey of HCMV in allogenic and autologous stem cell transplantation by real-time PCR in Kermanshah, west of Iran

**DOI:** 10.1186/s13027-021-00349-4

**Published:** 2021-02-02

**Authors:** Mehrdad Payandeh, Mohammad Hossein Zamanian, Bizhan Nomanpour, Mohammad Soroush Farhadi, Alireza Janbakhsh, Mosayeb Rostamian, Azam Elahi, Somayeh Jafari, Mohammad Dehghannejad

**Affiliations:** 1 Hematology and Medical Oncology Dept., Kermanshah University of Medical Sciences, Kermanshah, Iran; 2 Infectious Diseases Dept., School of Medicine, Kermanshah University of Medical Sciences, Kermanshah, Iran; 3 Microbiology Dept., Medical school of Kermanshah, Kermanshah University of Medical Sciences, Kermanshah, Iran; 4 Microbiology Dept., Islamic Azad University, Tehran North Branch, Tehran, Iran; 5 Infectious Diseases Research Center, Health Institute, Kermanshah University of Medical Sciences, Kermanshah, Iran; 6 Clinical Research Development Center, Imam Reza Hospital, Kermanshah University of Medical Sciences, Kermanshah, Iran; 7 Medical School of Kermanshah, Kermanshah University of Medical Sciences, Kermanshah, Iran

**Keywords:** Cytomegalovirus, Bone marrow transplant, Real-time PCR

## Abstract

**Introduction:**

Human Cytomegalovirus (HCMV) is the most important viral pathogen in people undergoing bone marrow transplantation (BMT). HCMV detection in the early stages makes is possible to save the patients’ lives through immediate and timely treatment. The aim of this study was to investigate the status of HCMV using the real-time PCR method in BMT patients in Kermanshah, west of Iran.

**Methods:**

HCMV monitoring was done in 120 patients who underwent BMT, 38 allogeneic cases and 82 autologous cases, using the ELISA serology test before transplantation. The participants were followed up 100 days after transplantation for HCMV detection in blood samples using real-time PCR. Preemptive therapy started with Ganciclovir and Foscarnet when the viral load was > 200 HCMV DNA copies/ml.

**Results:**

Despite preemptive therapy, infection recurred in less than 1 month. HCMV recurred more frequently in patients undergoing allogenic transplation versus those receiving autologous transplantation. Recurrence was seen in 5 patients receiving allogenic transplantation. HCMV recurrence occurred in five patients with allogeneic transplantation. Twelve patients undergoing allogeneic or autologous transplantation (83%) and a virus load of > 1000 copies/ml showed HCMV-related symptoms. Three patients died, two due to HCMV-related pneumonia and the other one due to a fungal infection.

**Conclusion:**

Real-time PCR may be a useful method for quantification and monitoring of HCMV recurrence and may be helpful in choosing more efficient HCMV preemptive treatment in BMT recipients.

## Introduction

Cytomegalovirus (HCMV) is a member of the herpes virus family. This virus can affect various cells of the host body including endothelial, epithelial, and hematopoietic cells [1]. Antibodies against HCMV have been identified in the serum of 80% of healthy adults, indicating previous infection and the latency of the virus that could be reactivated [2].

In transplant recipients, suppression of the immune system triggers HCMV reactivation by both primary infections (i.e. infection in patients that are not detectable or sero-negative before transplantation) or secondary infections (i.e. infections due to the activation of a latent infection) [3]. Primary infection may cause different morbidities and even mortality in severe forms. It has a higher frequency, more clinical manifestations, and a higher recurrence rate than secondary infections [4, 5].

Superinfection by HCMV occurs when a seropositive recipient (R+) receives cells from a seropositive donor (D+); therefore, the origin of HCMV is reactivated after transplantation [6].

Secondary and superinfections can lead to clinical manifestations of HCMV in approximately 30% of stem cell transplant recipients. Such patients usually show milder symptoms than those infected by primary infections [7]. Regardless of the previous seropositive status of the donor or the recipient, HCMV occurs in 30–50% of bone marrow allogeneic recipients, which may progress severe HCMV [8]. However, HCMV-related diseases are approximately two times more common in D+/R+ patients than in D+/R- patients, which may be due to coincidence of active secondary infection and superinfection after transplantation [6].

HCMV infection is usually seen in the late postoperative period [8]. The onset of HCMV disease is often reported in a period of about 28 to 72 days after transplantation. It involves several organs including the lungs and intestines [9]. HCMV is associated with various complications such as pneumonia, gastrointestinal infections, central nervous system infections, and retinitis as well as many other miscellaneous disorders such as cystitis, nephritis, myocarditis, and pancreatitis [10]. New HCMV infection in a patient with former manifestations of the infection who did not have HCMV detected during active surveillance for an interval of at least 4 weeks is called “recurrent infection”. Recurrent infection may be an outcome of reinfection (exogenous) or reactivation of the latent virus (endogenous) [10].

The strategy for HCMV treatment in transplantation is preemptive therapy, and various studies have shown that this strategy is preferable to public prophylaxis. Ganciclovir is usually used for preemptive therapy. Foscarnet is an alternative in drug resistant cases [11]. After transplantation, treatment should be initiated when the blood viral load reaches a significant level before the onset of clinical symptoms. Therefore, the HCMV load in the blood should be determined in this strategy [12, 13].

Monitoring the viral load is a valuable prognostic indicator for HCMV treatment and prevention [8, 14]. Several diagnostic methods are available for monitoring of the patients at risk for HCMV infection [8]. Serological methods are associated with false positive results and are only useful in the pre-transplant stage to detect the presence of anti-HCMV antibodies in donor and recipient individuals, as well as in blood transfusion [15]. The pp65 antigen test is another serological test that detects HCMV antigens on mononuclear cells in the peripheral blood. However, it has some limitations including being subjective, labor intensive nature of the test, relative lack of standardization, and lower sensitivity compared to PCR [8, 16].

Furthermore, culturing HCMV is a difficult and time-consuming process and since the transplanted patients take immunosuppressive drugs, the viral growth is overwhelming [17].

Nowadays, real-time PCR is considered the standard method for determining the quantity of HCMV in the blood of patients with immune deficiency. This method is used to measure the DNA load in different samples. Real-time RCR has some advantages such as higher sensitivity and a limited turnaround time; hence, it has replaced the pp65 antigen test in many health centers around the world [8]. The aim of this study was to investigate the viral load of HCMV in bone marrow transplant patients.

## Methods

### Patients

This cross-sectional study was performed in the bone marrow transplant ward of Imam Reza Hospital, Kermanshah Province, west of Iran from April 2017 to May 2020. In total, 120 patients receiving bone marrow transplantation including allogeneic stem cell transplantation (alloSCT) and autologous SCT (autoSCT) were included in this study.

### HCMV surveillance

Whole blood (3–5 ml) samples with EDTA as anticoagulant were collected from patients presenting to the bone marrow transplant ward. The status of HCMV was tested in the serum of all SCT recipients and donors before transplantation to detect HCMV antibody using an ELISA kit (Diapro Kit, Italy). ELISA is the most frequent serologic test for assessing antibodies against HCMV. An ELISA positive test for HCMV antibodies shows that a person has been infected with HCMV during life but it does not show the time of infection.

The patients were categorized into four groups based on seronegativity or seropositivity of the recipient and donor before transplantation as R-D-, R + D-, R-D+ and R + D+).

### DNA extraction and real-time PCR

Since HCMV-DNAemia is an independent risk factor for HCMV infection [18], virological surveillance for virus replication is routinely performed in the first 100 days post-transplant using real-time PCR for monitoring of HCMV-DNA in the blood samples.

Using 200 μl of each patient’s plasma sample, HCMV genomic DNA was extracted using QIAamp DNA mini kit (Qiagen, Germany) according to the manufacturer’s instructions. TaqMan®-MGB probed real-time PCR was applied using HCMV RQ PCR kit (Novin-Gene, Iran) according to the manufacturer’s instructions. The limit of detection (LOD) of the kit was 200 copies /ul.

The thermal cycling program started with 10 min at 95 °C for initial denaturation followed by 45 cycles of denaturation (15 s at 95 °C), annealing (60 s at 58 °C), and extension (30 s at 58 °C). The program was run in the ABI Step One Plus real-time PCR (Applied Biosystems, USA) in triplicate. To quantify HCMV loads, a standard curve was created using standards of the kit that contained 10,000, 1000, 100, and 10 virus DNA copies/ul.

The results were included when the efficiency (E) was more than 90% and R2 was more than 88%.

### Prophylactic strategies

Based on prophylactic or preemptive tests, preemptive therapy started when > 200 virus DNA copies/ul [19] were detected in the serum using 10 mg/kg Ganciclovir or 180 mg/kg Foscarnet once a day for 2–3 weeks. Then, Ganciclovir 5 mg/kg or Foscarnet 90 mg/kg was given daily until two consecutive negative viral loads were obtained.

### Ethical consideration

The Ethics Committee of Kermanshah University of Medical Sciences, Kermanshah, Iran approved the study (ethics code: IR.KUMS. 980,164). Written informed consent was obtained from all patients for participation in the study.

## Results

### Patients

Of the 120 patients receiving bone marrow transplants, 82 (68.3%) were male and 38 (31.6%) were female. The mean age of the patients was 47.7 ± 13.38 years. Fifty-six patients (46.6%) had multiple myeloma (MM), 24 patients (20%) had Hodgkin’s lymphoma, 21 patients (17.5%) had acute myeloid leukemia (AML), 10 patients (8.3%) had non-Hodgkin’s lymphoma, 6 patients (5%) had acute lymphocytic leukemia (ALL), and 3 patients (2.5%) had myelofibrosis (MF).

Thirty-six patients (30%) had underlying diseases such as hypertension, high blood sugar, and abnormal lipid profile. Moreover, 76 patients (63%) were sero-positive for HCMV. Thirty-eight patients (31.6%) received allogeneic transplantation and 82 (68.3%) received autologous transplantation (Table 1).

**Table 1:** Patient characteristics

Variables	Frequency	Percent
**Sex**		
**Male**	82	68.3%
**Female**	38	31.6%
**Underlying disease**	36	30%
**Type of bone marrow transplant**		
**autoSCT**	82	68.3%
**alloSCT**	38	31.6%
**HCMV**		
**Serostatus**	21	17.5%
**real time PCR**	28	23.3%
**Death**	3	2.5%
**sum**	120	100

### AlloSCT patients – HCMV recurrence

In the follow-up period (median 100 days after transplantation), HCMV DNA was detected in 23 (60%) cases. The time between the transplantation day and the day of first HCMV positive detection was defined as time to infection (TTI). The median TTI was 27.9 days (range: 4 to 100 days) and the median viral load was 2.4× 10^4^ copies/ul (range: 23 to 3.3 × 10^5^ copies/ul). HCMV recurrence was observed in five patients (21.7%) that were high risk for HCMV recurrence, three of whom had AML, one had relapsed MM and one had relapsed MF (Table 2). HCMV recurrence occurred at least 2 to 3 weeks after transplantation.

**Table 2:** Patient characteristics (alloSCT patients) with positive HCMV real-time PCR result

Patient No.	Age and Sex	Disease	Donor	Underlying disease	Day of first positive RT-PCR result	Peak HCMV level (copies/ml)	Recurrence	HCMV Serostatus (D/R)	GVHD	Outcome
1	42 M	AML	Sister	+	38	88,600 ± 15	–	−/ -	–	Alive (Hematuria)
2	32 M	ALL	Mud	–	23	4000 ± 8	–	−/+	–	Alive (Liver disorders)
3	48 M	MM	Brother	–	96	64 ± 1	–	+/ +	–	Alive
4	56 M	AML	Brother	–	10	230 ± 2	+	+/ +	Skin	Alive
5	40 M	AML	Brother	–	4	835 ± 1	–	+/ +	–	Alive
6	33F	ALL	Brother	–	4	480 ± 2	–	−/ -	GI	Alive
7	41 M	ALL	Brother	+	10	563 ± 3	–	−/ +	Skin	Alive
8	42 M	ALL	Brother	–	100	670 ± 1	–	+/ +	Compound	Alive
9	21 M	AML	Brother	–	40	120 ± 1	–	−/ +	Compound	Alive
10	37 M	AML	Brother	–	7	300 ± 1	–	+/ +	–	Alive
11	26 M	AML	Brother	–	28	117,413 ± 16	–	+/ +	–	Alive (Diarrhea)
12	50 M	AML	Mud	+	56	580 ± 1	+	−/ +	Compound	Alive
13	46 M	AML	Sister	–	30	4320 ± 3	–	−/ +	–	Died Of Fungal Complications
14	53 M	MF	Mud	+	9	8310 ± 11	+	−/ +	Compound	Alive (CNS disorders)
15	57F	AML	Mud	+	12	315 ± 2	–	−/ +	Compound	Alive
16	39 M	AML	Brother	–	8	23 ± 0	–	+/ +	Skin	Alive
17	46F	ALL	Brother	+	19	191 ± 0	–	+/ +	GI	Alive
18	46F	AML	Mud	–	18	325,967 ± 25	+	+/ +	GI	Alive (Pneumonitis)
19	58F	MM	Mud	–	38	6174 ± 13	–	+/ +	Skin	Alive (Digestive disorders)
20	30 M	MM	Sister	+	11	39 ± 0	–	−/ -	–	Alive
21	31F	MM	Mud	–	26	6579 ± 5	+	+/ +	GI	Alive (Hemorrhagic cystitis, Kidney failure)
22	55F	NHL	Sister	–	25	452 ± 1	–	+/ -	–	Dead (Pneumonitis)
23	67 M	AML	Sister	–	31	250 ± 2	–	−/ -	–	Alive

*MUD* Matched Unrelated Donor, *R-* Recipient HCMV negative, *D-* Donor HCMV negative, *R+* Recipient HCMV positive, *D+* Donor HCMV positive, *HCMV* Cytomegalovirus, *AML* Acute Myeloid Leukemia, *CML* Chronic Myeloid Leukemia, *ALL* Acute Lymphoblastic Leukemia, *MM* Multiple Myeloma, *HL* Hodgkin Lymphoma, *NHL* Non-Hodgkin Lymphoma, *MF* Myelofibrosis, *GVHD* Graft-Versus-Host Disease, *PCR* Polymerase Chain Reaction

In alloSCT patients, HCMV led to kidney diseases such as cystitis or hematuria (10 cases), gastrointestinal disorders such as diarrhea (2 cases), pneumonia (2 cases), liver disorders (2 cases), CNS disorders (1 case) and fungal infections (1 case). The viral load was > 1000 copies/ul in 9 of these patients. Only one case had a lower viral load that died from fungal infections. The mortality rate was 2 cases (8.6%) in 100 days of follow-up.

### AutoSCT patients – recurrence of HCMV

Of 82 autoSCT patients, 51cases (62%) were HCMV seropositive, indicating previous exposure to the virus. HCMV DNA was detected in 5 (6%) cases. The median TTI in these patients was 10 days (range: 1 to 26 days) and the median viral load was 688.8 DNA copies/ul (range: 50 to 2.3× 10^3^ DNA copies/ul). No HCMV recurrence was observed in any of the autoSCT patients (Table 3).

**Table 3:** Patient characteristics (autoSCT patients) with positive HCMV real-time PCR result

Patient No.	Age and Sex	Disease	Underlying disease	Day of first positive RT-PCR result	Peak HCMV level (copies/ml)	Recurrence	HCMV Serostatus	Outcome
1	49 M	HL	–	26	846 ± 3	–	+	Dead (Pneumonitis)
2	51F	MM	–	4	50 ± 0	–	–	Alive
3	63 M	MM	–	17	2366 ± 4	–	+	Alive (Colitis and Diarrhea)
4	67F	MM	–	2	125 ± 1	–	–	Alive
5	55F	NHL	–	1	57 ± 0	–	+	Alive

Two patients with a viral load of > 200 DNA copies/ul developed HCMV-related complications, including pneumonia and gastrointestinal disorders (colitis and diarrhea). One of these patients did not respond well to treatment and died, but the other one responded to treatment and survived.

## Discussion

Although results of studies investigating HCMV complications in bone marrow stem cell recipients are very challenging, significant progress has been made in reducing the clinical effects of the infection in low- to moderate-risk recipients. However, the recipients are still at risk for HCMV infection and its indirect effects due to severe suppression of the immune system [20].

Quantitative measurement of HCMV in the blood by real-time PCR is very helpful in diagnosing HCMV, predicting the prognosis of HCMV before symptoms occur, and evaluating response to treatments [21]. Early detection of HCMV infection leads to prophylaxis or preventive prescription earlier than treatment, which can reduce both the severity of HCMV complications and HCMV-related mortality [22, 23]. However, despite advances in HCMV prevention, it is still an important cause of mortality in immune suppressed patients [24].

HCMV infection can be in the form of primary infection, virus reactivation, or superinfection. In case of bone marrow transplantation, the serological status of the recipient is a significant risk factor for HCMV reactivation, transplantation rejection, and the recipient’s mortality [25–27].

In the present study, in low-risk allograft cases where both donors and recipients were ELISA negative (R−/D-) before transplantation, real-time PCR evidence showed HCMV contamination of the recipients after transplantation. Similarly, Butt et al. reported that 55% of their low-risk (R−/D-) patients were HCMV positive after transplantation according to real-time PCR results [4].

The origin of HCMV is unclear in these cases, but it may be transmitted by blood products after transplantation. Moreover, the donor or the recipient may already have a HCMV infection but be a weak producer of HCMV antibodies, so they may be misdiagnosed as HCMV negative cases using techniques that rely on antibody detection such as ELISA. Many bone marrow transplantation patients are suppressed by previous chemotherapy, which may lead to a decrease in HCMV antibody titer and misdiagnosis of an R+ patient as a low-risk recipient.

The risk of HCMV reactivation has been also shown in autologous bone marrow transplantation where seronegative patients are infected with HCMV after transplantation. This condition is mainly observed when methods other than RT-PCR are used. Most studies using RT-PCR suggest a percentage of post-ASCT HCMV DNA infections of about 9.1–17.6% [28–31], which is more frequently observed in HCMV IgG seropositive patients [30]. In our study, only 6% of the patients had a post-ASCT HCMV DNA infection, which is lower than the average values reported in other studies. The reason for this discrepancy is not known but it may be related to differences in experimental settings such as sample size, materials and methods used, etc.

Previous studies reported an incidence of 0–22% for HCMV infection in transplantation patients [32]. There are also other factors involved in HCMV infection in these patients such as heavy diets, type of medications, etc. [33, 34] Attention to these factors may be helpful in HCMV screening of all bone marrow transplantation patients at the initial diagnosis steps.

HCMV infection is usually asymptomatic, but it sometimes occurs in some patients in the form of a serious and fatal infection. The most common manifestations of HCMV infection are pneumonia and gastrointestinal disease (which may involve any organ from the esophagus to the large intestine) [20]. In our study, HCMV caused disease in 12 cases with the most common symptoms being pneumonia and gastrointestinal disorders. Three patients died, two from pneumonia and one from a fungal infection. Previous studies have shown that most HCMV-related deaths were due to bacterial and fungal sepsis, confirming an association between HCMV and other opportunistic infections. The incidence of graft versus host disease (GVHD) is also indirectly associated with HCMV infection [20].

One of the most important factors in predicting lack of response to HCMV antiviral therapy is the pre-treatment viral load. The optimal threshold of the viral load to start antiviral therapy in hematopoietic cell transplant (HCT) recipients has not yet been determined [35]. Our findings are in line with previous reports in which HCMV treatment has been associated with shorter viremia episodes and shorter courses of antiviral therapy [36, 37]. High-risk allogeneic recipients require longer exposure to antiviral therapy because HCMV is often present in a longer period of time in these patients [35]. A study by Camargo et al. showed that antiviral therapy in high-risk patients with an antibody level of ≥150 IU/mL should not be delayed for the first 100 days after transplantation [35]. Furthermore, Green et al. found that early initiation of antiviral therapy was not only useful but also well tolerated by the patients [28]. Green et al. reported an association between a viral load of ≥250 IU/mL and increased risk of mortality in the early postoperative period [38]. Camargo et al. also found a high probability of asymptomatic mortality in patients who failed to eradicate the HCMV virus [35].

Identifying the cutoff level for real time PCR test could be an important indicator of the proper time to start anti-HCMV treatment [39]. Using real time PCR, Mhiri et al. determined a cut-off value of above 400 DNA copies/ml in plasma samples [40].

Garrigue et al. and Ghaffari et al. reported a cut-off value of 6300 and 1000 DNA copies/ml for HCMV infection using real-time PCR, respectively [28, 41]. However, it is not possible to establish a universal cut-off value for the initiation of anti-HCMV treatment and it seems that the cut-off value should be confirmed by testing many patients in each study [39].

According to a study by Mhiriet al., the initial load of HCMV is significantly associated with the progression of the disease [40]. Weinberg et al. found that patients with recurrent HCMV were still positive for real-time PCR at the time of treatment discontinuation, while patients who did not experience recurrence tended to clear HCMV faster [42].

Numerous studies have shown that the risk of developing HCMV is directly associated with the viral load [35, 38]. However, in the present study, even patients with low viral loads developed HCMV infection, emphasizing the importance of early initiating of antiviral therapy.

In the present study, 71% of HCMV positive cases had a viral load of above 2 × 10^2^ DNA copies/mL and HCMV recurrence was seen in 21% of the patients. Furthermore, 83% of the recipients were seropositive and two of them died, one with less than 1000 HCMV DNA copies/ml. Similar to our results, Banon et al. found HCMV-related diseases in three cases (8.7%) with less than 1000 HCMV DNA copies/ml [43]. In addition, several cohort studies have shown that HCMV presence or reactivation is associated with an increased risk of asymptomatic mortality [44, 45].

In the present study, the time between the transplantation and first detection of HCMV DNA was about 1 month in allogeneic transplant cases and about 10 days in autologous transplant cases. This indicates that due to immunosuppression, HCMV infection is more common in the first 3 months after transplantation, which is in accordance with previous reports [46].

Due to the limited availability of antiviral drugs, preemptive therapy has become the standard of care for the prevention of HCMV following blood cell transplantation [35]. Real-time PCR can be used as a potential method for screening patients at regular intervals, and if a positive result is observed, the sequential evaluation process should begin. This strategy can lead to much earlier antiviral treatment. Therefore, attention should be paid to the viral load by regular check-ups of the patients, even when their viral load is negative, to effectively control this disease.

## Conclusion

This study confirms the importance of viral load in the pathogenesis of HCMV after stem cell transplantation. Quantitative real-time PCR may be a useful tool for monitoring HCMV recurrence and response to antiviral therapy in bone marrow transplant recipients. The high incidence of seropositive HCMV in bone marrow transplant candidates confirms the need for an appropriate program to prevent HCMV-related complications through early diagnosis and treatment.
